# Sulfated Polysaccharides of *Potamogeton lucens* as a Promising Immunostimulatory Agent in Activation of RAW264.7 and NK Cells

**DOI:** 10.1002/fsn3.70692

**Published:** 2025-08-08

**Authors:** Rongan Cao, Mehdi Tabarsa, SangGuan You, Fatemeh Noormand Chaloshtori, Jiamiao Zhang, Kou Fang, Seyedeh Zahra Bathaie

**Affiliations:** ^1^ College of Food Science Heilongjiang Bayi Agricultural University Daqing China; ^2^ Department of Seafood Processing Faculty of Marine Sciences, Tarbiat Modares University Noor Iran; ^3^ Bioactive Compounds Group Faculty of Interdisciplinary Sciences and Technologies, Tarbiat Modares University Tehran Iran; ^4^ Institute for Natural Products and Medicinal Plants Tarbiat Modares University Tehran Iran; ^5^ Department of Marine Food Science and Technology Gangneung‐Wonju National University Gangneung Gangwon Republic of Korea; ^6^ Department of Clinical Biochemistry Faculty of Medical Sciences, Tarbiat Modares University Tehran Iran

**Keywords:** freshwater plant, immunostimulatory, structure, sulfated polysaccharide

## Abstract

Sulfated polysaccharides are naturally occurring biomacromolecules with complex structures and promising therapeutic effects, the mechanism of which remains to be fully understood. This study isolated a novel sulfated polysaccharide from *Potamogeton lucens* , followed by fractionation by a DAEA Sepharose FF column to assess its immunostimulatory effects on NK‐92 and RAW264.7 cells. The primary components within the crude polysaccharides and fractions (PLF1, PLF2 and PLF3) were predominantly neutral sugars, with comparatively lower proportions of sulfate and uronic acids. The isolated polysaccharides exhibited a range of weight average molecular weights (*M*_w_) from 488.7 to 918.9 × 10^3^ g/mol. Polysaccharide composition included glucose, galactose, arabinose, rhamnose, mannose, and xylose units linked through various glycosidic linkages, including (1 → 4)‐Gal*p*, (1 → 6)‐Gal*p*, (1 → 3)‐Gal*p*, (1 → 3)‐Ara*p*, (1 → 2,4)‐Rha*p*, (1 → 2)‐Glc*p* and (1 → 4)‐Glc*p* residues. The PLF2 polysaccharide showed remarkable efficacy by activating RAW264.7 macrophage cells to synthesize NO, TNF‐α, IL‐1β and IL‐6, while also activating NK‐92 cells to synthesize TNF‐α, INF‐γ, granzyme‐B, perforin, NKG2D, and FasL via the NF‐κB and MAPKs signaling pathways. Collective findings indicated that polysaccharides derived from *P. lucens* have the potential to serve as potent immunostimulatory compounds in functional foods, capable of eliciting responses in both RAW264.7 and NK cells.

## Graphical abstract

The polysaccharide showed remarkable efficacy by activating RAW264.7 macrophage cells to synthesize NO, TNF‐α, IL‐1β and IL‐6, while also activating NK‐92 cells to synthesize TNF‐α, IFN‐γ, granzyme‐B, perforin, NKG2D, and FasL via the NF‐κB and MAPKs signaling pathways.

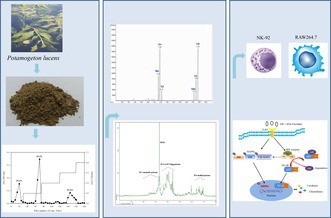

## 1 Introduction

Anionic polysaccharides, due to the presence of carboxyl and sulfate groups, can be naturally found in various marine organisms, particularly brown, red, and green seaweeds. These marine‐derived polysaccharides are characterized by a high level of complexity in the monosaccharide contents that build up the polymer structure (Cho et al. 2014; Tabarsa et al. 2018). Galactans, including agar and carrageenan, are found in the cell wall matrix of red seaweeds and consist of L‐ and D‐galactopyranose residues (Campo et al. 2009). Ulvans, on the other hand, are polysaccharides extracted from green seaweeds and are primarily composed of α‐L‐rhamnose monosaccharides (Tabarsa, Han, et al. 2012; Tabarsa, Lee, and You 2012; Rahimi et al. 2016). Another group of anionic macromolecules, known as fucose‐containing polysaccharides, is found not only in brown seaweeds but also in sea urchins and sea cucumbers (Cao et al. 2017; Borazjani et al. 2017). It is necessary to highlight that the synthesis of sulfated polysaccharides is not limited to marine habitats, as sulfate esters have also been found in the polysaccharide chains of *Hydrocotyle bonariensis, Myriophyllum spicatum*, *Eichhornia crassipes, Cryptomonas obovate*, and *Nymphaea ampla* (Giroldo and Vieira 2002; Dantas‐Santos et al. 2012; Alavi et al. 2020). The diverse array of biological activities attributed to sulfated polysaccharides is believed to be in close relation to structural characteristics such as sugar content, glycosidic linkages, size of the molecule, carboxyl and sulfate groups, either individually or collectively (Ferreira et al. 2015; Borazjani et al. 2017; Chaloshtori et al. 2023).

Modulation of the immune system represents a noteworthy biological impact of sulfated polysaccharides. When stimulated, this regulation can either be enhanced to release pro‐inflammatory mediators or suppressed to manifest an anti‐inflammatory response. Human immunity comprises diverse biological elements and processes that are proficient in discriminating between body cells and foreign pathogens within both the innate and acquired immunity subsystems (Fontana et al. 2018; Mosser and Edwards 2008). The innate immune system is the first line of defense of the body and can react quickly to foreign agents without needing to have seen them before, starting an inflammatory response right away (Mosser and Edwards 2008). Subsequent to the initial contact, macrophages and NK cells execute protective measures against microbes and abnormal cells via diverse mechanisms, encompassing the release of NO, exocytosis of secretory lysosomes, and stimulation of other immune cells (Topham and Hewitt 2009). Despite the fact that full activation of the immune system is necessary in eliminating malignant cells and pathogens, its efficacy is compromised in cancers and infections that afflict patients with autoimmune diseases, attributable to the production of immunosuppressive factors (Ferreira et al. 2015). Therefore, the strategic application of immunostimulatory agents is of paramount importance in therapeutic interventions for cancers and infections. Hence, the current investigation is designed to study the structural analysis of sulfated polysaccharides derived from *Potamogeton lucens* . Additionally, the study aims to evaluate its potential to activate immune cells, providing a detailed examination of the mechanisms underlying their actions.

## 2 Materials and Methods

### 2.1 Samples and Reagents

Raw *Potamogeton lucens* specimens were gathered from Sarab‐e‐Ravansar, Kermanshah, Iran. The plant samples were subjected to a thorough cleaning process involving the use of seawater to eliminate any residual materials. Subsequently, the plants were further cleaned with running water and kept in an oven (60°C) to dry. The dried plants were then finely ground and stored at a temperature of −20°C. Monosaccharide standards, NaBD_4_, methyl iodide (CH_3_I) and trifluoroacetic acid were purchased from Sigma‐Aldrich (St. Louis, MO, USA). The DC protein assay kit used in this study was from Bio‐Rad (CA, USA). Other chemicals utilized in the present investigation were of analytical grade.

### 2.2 Extraction and Fractionation of Polysaccharides

**FIGURE 1:**
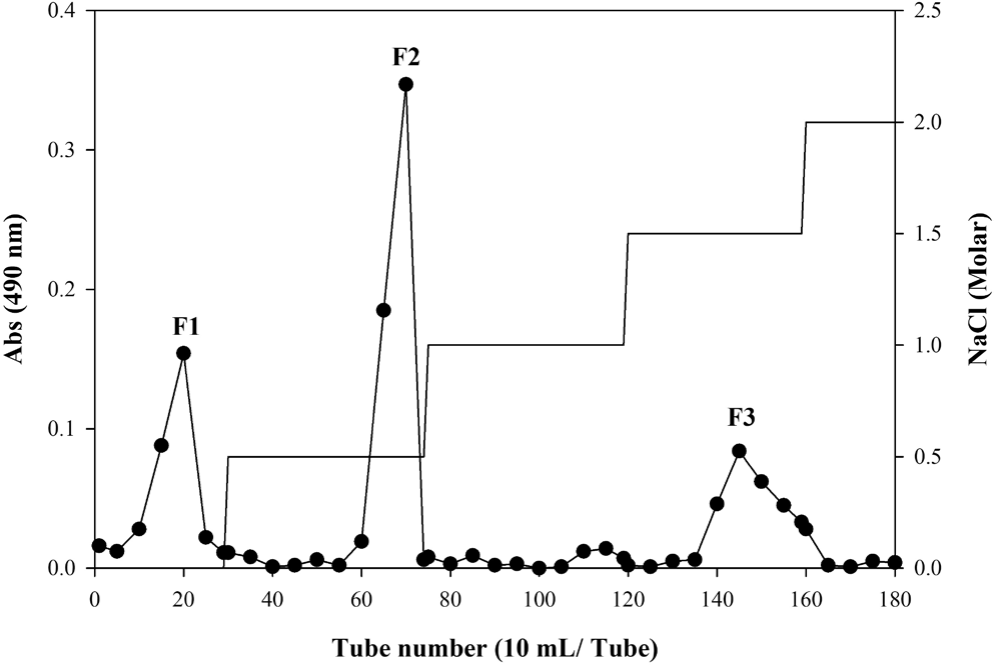
The chromatographic elution profile of polysaccharides was observed on a DEAE Sepharose Fast Flow column.

The sample powder soaked in 80% ethanol at ambient temperature to eliminate undesired compounds and secondary metabolites. The centrifugation process was then performed o at a temperature of 10°C and a gravitational force of 6080 *g* for a period of 10 min to divide the liquid phase. The residue was subsequently subjected to acetone washing and kept at room temperature for a total of 24 h to dry. The polysaccharide was extracted from the defatted powder (5 g) using distilled water at 65°C for a duration of 2 h. The supernatant was obtained following centrifugation (10°C and 6080 *g* for 10 min) and evaporated under reduced pressure. Polysaccharides were then isolated from the solution through the use of 99% ethanol in a ratio of 1:3 (v/v). The polysaccharide was subjected to a series of ethanol and acetone washing steps. The crude polysaccharide, denoted PLP, was air dried at ambient temperature. Subsequently, 250 mg of PLP was dissolved in water (10 mL) which was followed by filtration (3.0 μm) and loading onto a DEAE Sepharose FF column. The column underwent initial elution with distilled water, and subsequently a gradual gradient of 0.5–2.0 M NaCl was employed for sample washing. The eluents were analyzed by phenol‐sulfuric acid method and fractions positive for carbohydrates were combined (DuBois et al. 1956), dialyzed and then lyophilized, resulting in the generation of three fractions named PLF1, PLF2, and PLF3 (Figure 1).

### 2.3 Chemical Analysis

Total neutral sugars (Appendix S1) were measured by the phenol‐sulfuric acid method, with D‐glucose serving as a reference (DuBois et al. 1956). The sulfamate/m‐hydroxydiphenyl method was used to quantify the amount of uronic acid (Appendix S1) with glucuronic acid serving as a reference (Filisetti‐Cozzi and Carpita 1991). The level of sulfate (Appendix S1) was measured by the BaCl_2_‐gelatin method, with K_2_SO_4_ serving as a reference (Dodgson and Price 1962). Protein quantification (Appendix S1) was carried out using the Lowry method and the DC protein assay kit from Bio‐Rad (USA) using bovine serum albumin as a reference (Lowry et al. 1951).

### 2.4 Determination of Monosaccharides and Glycosidic Linkages

As suggested by Ciucanu and Kerek (1984), polysaccharides (2 mg) underwent the process of methylation using CH_3_I in DMSO and/or hydrolysis using 0.5 mL of 4 M TFA, which lasted for 6 h at 100°C. The TFA was subsequently evaporated with a flow of nitrogen, followed by adding distilled water (0.5 mL) and NaBD_4_ (5 mg) in order to produce reduced derivatives. The process was halted by the introduction of acetic acid, and then the mixture was subjected to further drying under a stream of nitrogen while being heated. Ultimately, acetic anhydride was introduced into the sample solution and maintained at 100°C for 2 h, leading to the production of acetylated products. The ultimate derivatives were analyzed using a gas chromatography mass spectrometry (GC–MS) system (6890 N/MSD 5973, Agilent Technologies, Santa Clara, CA) as described previously (Shemami et al. 2018).

### 2.5 ATR‐FTIR and ^1^HSpectrometrieseries

Samples (20 mg) were dispersed in D_2_O and analyzed using a JEOL ECA‐600 spectrometer (JEOL, Akishima, Japan) at a temperature of 50°C to obtain the ^1^H NMR spectrum. The polysaccharide was subjected to analysis using ATR‐FTIR in transmittance mode within the range of 600 to 4000 cm.^−1^ (Tensor 27, Bruker Instruments, Billerica, USA).

### 2.6 Molecular Weight Analysis

Polysaccharides were dissolved in water (2 mg/mL) and filtered using a cellulose membrane (3.0 μm) (Whatman International). The molecular weight characteristics including weight average molecular weight (*M*_w_), the radius of gyration (*R*_g_) and specific volume of gyration (*SV*_g_) were obtained by a HPSEC‐MALLS‐RI system (Technology Corp, Santa Barbara, CA, USA HELEOS; Wyatt) as described previously (You and Lim 2000; Chaloshtori et al. 2023).

### 2.7 Proliferation of RAW264.7 Murine Macrophage Cells

The effect of polysaccharides on RAW264.7 cells was evaluated as described previously by Chaloshtori et al. (2023). In brief, RAW264.7 cell lines were grown under controlled conditions of 37°C and 5% CO_2_ in culture medium composed of RPMI‐1640 and 10% FBS. Then, cell suspension (100 μL) was subsequently placed in 96‐well microplates at a density of 1 × 10^5^ cells/mL. Following a 24‐h incubation time, polysaccharides at different concentrations (10–50 μg/mL) were introduced into the wells (100 μL per well) and the plates were incubated for 24 h. The culture medium was discarded, and 120 μL of WST‐1 reagent was used to incubate with the cells for 4 h. The optical density was recorded at 450 nm using a microplate reader.

### 2.8 Activation of RAW264.7 Cells to Secret Nitric Oxide

As an indication of activated RAW264.7 cells, the amount of nitric oxide was measured as previously described by Chaloshtori et al. (2023). In brief, culture medium composed of RPMI‐1640 and 10% FBS was employed to culture RAW264.7 cells at a temperature of 37°C under 5% CO_2_. The cells were placed in a microplate and incubated with polysaccharides (10 to 50 μg/mL) or LPS derived from *Escherichia coli* (1 μg/mL) for 24 h. The culture medium was combined with Griess reagent, and optical density was measured at 540 nm (Green et al. 1982).

### 2.9 Gene Expression Analysis

Culture medium composed of RPMI‐1640 and 10% FBS was employed for the cultivation of RAW264.7 cells (1 × 10^5^ cells/well). Cells were treated with polysaccharides (10 to 50 μg/mL) for 18 h at 37°C. The MEM medium containing 10% fetal bovine serum, 10% horse serum, 0.2 mM inositol, 0.02 mM folic acid, and 0.1 mM β‐mercaptoethanol was utilized for the cultivation of NK‐92 cells, wherein varying concentrations of polysaccharides (10–50 μg/mL) were subsequently included and kept for a period of 24 h at 37°C. The cells were collected, and the total mRNA content of either RAW264.7 or NK‐92 cells was isolated using TRIzol reagent (Invitrogen, Carlsbad, CA, USA) following the manufacturer's protocol. The cDNA of the isolated mRNA was synthesized using oligo‐(dT)_20_ primer and Superscript III RT (Invitrogen, Carlsbad, CA, USA). The PCR amplification was conducted using GoTaq Flexi DNA Polymerase (Promega, Madison, WI, USA) and specific primers (Table 1) with the following conditions: 30 cycles of 94°C for 30 s, 56°C for 40 s, and 72°C for 1 min, followed by a final step of 72°C for 10 min. The PCR products were electrophoresed on a 1% agarose gel and stained with ethidium bromide. The gels were examined under a UV transilluminator (Kodak Digital Science, Kennesaw, GA, USA) and the results were expressed as the relative intensity compared to that of β‐actin (Alavi et al. 2020). The specific primers are presented in Table 1.

**TABLE 1:** Sequences of specific primers used for RT‐PCR.

Cells	Gene	Primer sequences (5′→3′)
RAW264.7	iNOS	(F)CTGCAGCACTTGGATCAGGAACCTG;(R)GGGAGTAGC CTGTGTGCACCTGGAA
	IL‐1β	(F)ATGGCAACTATTCCAGAACTCAACT;(R)CAGGACAGGTATAGATTCTTTCCTTT
	IL‐6	(F)TTCCTCTCTGCAAGAGACT;(R)TGTATCTCTCTGAAGGACT
	TNF‐α	(F)ATGAGCACAGAAAGCATGATC;(R)TACAGGCTTGTCACTCGAATT
	IL‐12	(F)CCACAAAGGAGGCGAGACTC;(R)CTCTACGAGGAACGCACCTT
	β‐Actin	(F)ATGTGCAAAAAGCTGGCTTTG;(R)ATTTGTGGTGGATGATGGAGG
NK‐92	TNF‐α	(F) CCTTGGTCTGGTAGGAGACG;(R)CAGAGGGAAGAGTTCCCCAG
	INF‐γ	(F)GATGCTCTTCGACCTCGAAACAGCAT;(R)ATGAAATATACAAGTTATAATCTTGGCTTT
	Granzyme‐B	(F)AGATCGAAAGTGCGAATCTGA;(R)TTCGTCCATAGGAGACAATGC
	Perforin	(F)AGTCCTCCACCTCGTTGTCCGTGA;(R)AAAGTCAGCTCCACTGAAGCTGTG
	NKG2D	(F)GACTTCACCAGTTTAAGTAAATC;(R)CTGGGAGATGAGTGAATTTCATA
	FasL	(F)CCAGAGAGAGCTCAGATACGTTGAC;(R)ATGTTTCAGCTCTTCCACCTACAGA
	β‐Actin	(F)CATCTCTTGCTCGAAGTCCA;(R)ATCATGTTTGAGACCTTCAACA

### 2.10 Western Blot Analysis

RIPA buffer that consisted of 50 mM Tris–HCl (pH 7.4), 150 mM NaCl, 1% Nonidet P‐40, and 0.1% sodium dodecyl sulfate was employed to lyse RAW264.7 macrophage cells or NK‐92 cells. The concentration of proteins was assessed using the Pierce BCA Protein Assay kit from Thermo Fisher Scientific. An equivalent amount of cell lysates containing 30 μg protein was subjected to electrophoresis on an SDS‐PAGE and subsequently transferred onto a polyvinylidene fluoride (PVDF) membrane. The membrane was then subjected to incubation with primary antibodies including anti‐phospho‐NF‐κB p65 (ab76302), anti‐phospho‐JNK (ab124956), anti‐phospho‐ERK1/2 (ab214362) and anti‐phospho‐p38 (ab4822, Abcam, Cambridge, United Kingdom). Following washing, the membrane was incubated with HRP‐conjugated anti‐rabbit antibody for a duration of 1 h at 4°C. The detection of proteins was accomplished using the Pierce ECL Plus Western Blotting Substrate from Thermo Fisher Scientific. The Bio‐Rad image analysis system was used to visualize the protein bands, and the software Quantity One (version 4.6, Bio‐Rad, USA) was utilized to quantify the protein expressions.

### 2.11 Statistical Analysis

All experiments in the current study were conducted in triplicates, and the outcomes are expressed as the mean value along with the standard deviation. Statistical comparisons among the test groups were performed using one‐way analysis of variance (ANOVA) in conjunction with Duncan's multiple range test. Statistical analysis was carried out using SPSS software (version 16; SPSS Inc., Chicago, IL, USA). Statistical significance was considered at a probability value of *p* < 0.05.

## 3 Results and Discussion

### 3.1 Chemical Analysis and Monosaccharides of Fucoidans

Polysaccharides of *P. lucens* (PLP) were extracted by water at high temperature. Table 2 shows the chemical composition of unrefined and purified polysaccharides. PLP yielded 7.4%, which was higher than those reported in the range of 1.7% to 7.0% for fucoidans from brown seaweeds (Ale et al. 2012; Jin et al. 2014) and lower than those reported in the range of 8.0% to 18.3% for polysaccharides from green seaweeds (Chattopadhyay et al. 2007; Rahimi et al. 2016). It should be noted that the amount of polysaccharides extracted from freshwater plants was found to be 3.0% for a galactofucan from *Azolla filiculoides* and 6.4% for a sulfated polysaccharide from *M. spicatum* (Shemami et al. 2018; Alavi et al. 2020).

**TABLE 2:** Chemical and monosaccharide compositions of native fucoidan and hydrolysates from *P. lucens* .

	PLP	PLF1	PLF2	PLF3
Yield	7.4	26.3	55.4	18.2
Neutral sugars	63.32 ± 1.11^d^	84.11 ± 1.21^a^	80.68 ± 1.61^b^	67.82 ± 0.80^c^
Sulfate	25.80 ± 0.24^a^	0.92 ± 0.13^d^	9.76 ± 0.27^c^	19.78 ± 0.79^b^
Uronic acids	3.68 ± 0.05^a^	2.07 ± 0.07^b^	4.23 ± 0.70^a^	1.65 ± 0.77^b^
Protein	4.03 ± 0.44^b^	0.7 ± 0.44^c^	0.64 ± 0.34^c^	7.58 ± 0.41^a^
Rhamnose	2.95 ± 0.21^b^	3.15 ± 0.49^b^	10.10 ± 0.84^a^	10.00 ± 1.55^a^
Arabinose	13.80 ± 0.84^c^	6.35 ± 0.77^d^	18.35 ± 0.63^a^	17.00 ± 1.13^b^
Xylose	4.80 ± 0.28^a^	2.85 ± 0.77^b^	3.35 ± 0.21^b^	2.85 ± 0.49^b^
Mannose	3.45 ± 0.35^c^	3.40 ± 0.42^c^	5.75 ± 0.07^b^	9.45 ± 0.63^a^
Glucose	55.50 ± 0.70^b^	70.85 ± 0.77^a^	17.35 ± 0.63^d^	23.85 ± 0.35^c^
Galactose	19.50 ± 0.56^c^	13.35 ± 0.77^d^	45.05 ± 1.06^a^	36.90 ± 0.98^b^
*M*_*W*_ × 10^3^ (g/mol)	881.8 ± 9.5	556.8 ± 10.0	918.9 ± 7.3	488.7 ± 5.0
*R*_g_ (nm)	82.5 ± 1.3	59.1 ± 0.7	61.5 ± 1.2	72.1 ± 1.5
*SV*_g_ (cm^3^/g)	1.63	0.93	0.64	1.94

*Note:* Weight average molecular weight (*M*_w_), radius of gyration (*R*_g_) and specific volume of gyration (*SV*_g_). Letters a, b, c, d denote significant differences (*p* < 0.05) between samples.

The PLP predominantly consisted of neutral sugars (63.3%), acidic sugars (3.7%) and sulfate esters (25.8%) (*p* < 0.05). The PLP extract contained a minimal amount of protein. As stated above, in addition to seaweeds, substituted polysaccharides with sulfate esters are also found in freshwater plants and algae (Giroldo and Vieira 2002; Dantas‐Santos et al. 2012). Biologically, the synthesis of polysaccharides containing sulfates in plants and algae has been proposed to link with water salinity and stressful conditions such as extended desiccation periods, changes in ionic strength, and exposure to high temperature (Arata et al. 2017).

**FIGURE 2:**
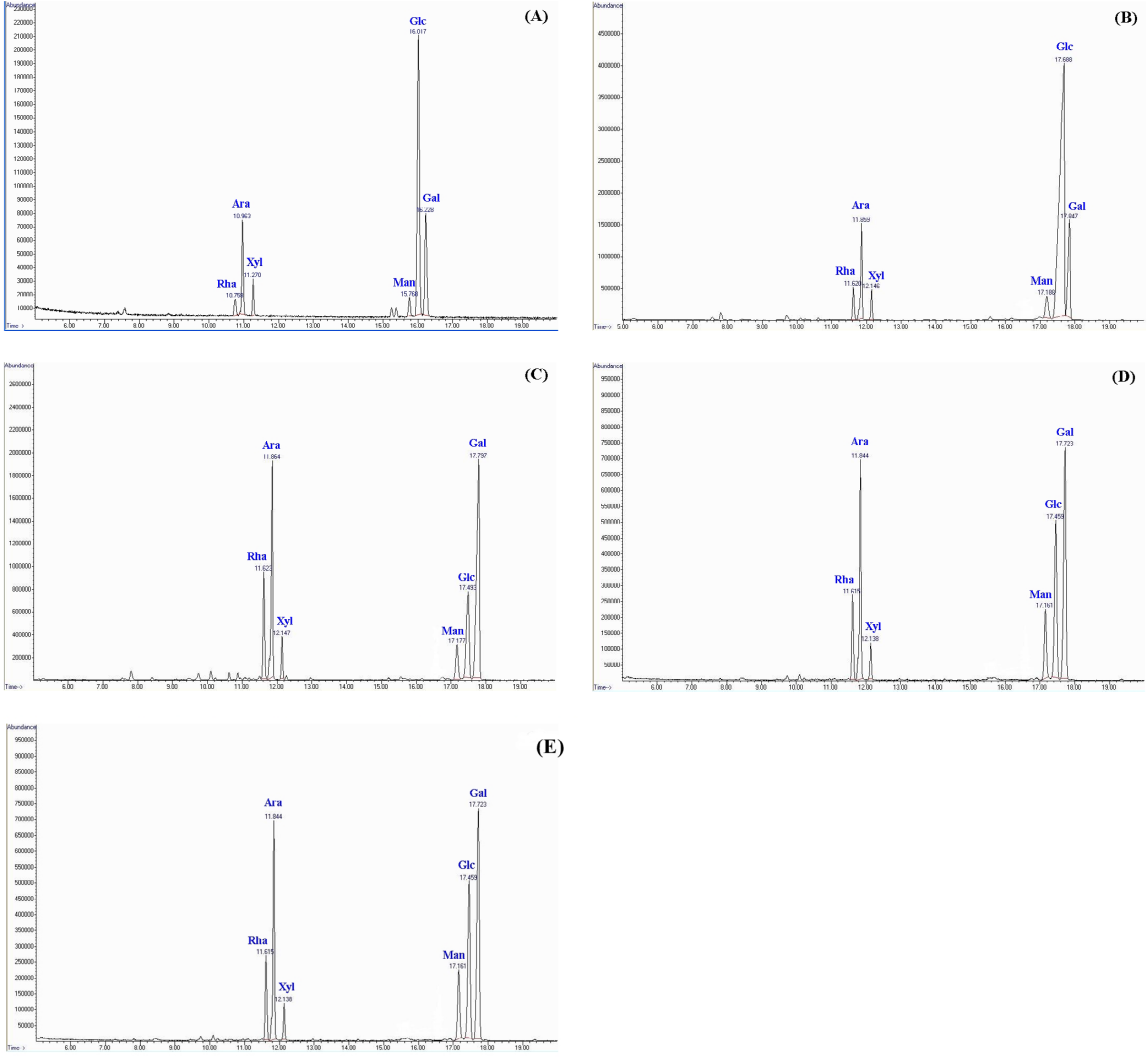
Gas chromatography (GC) chromatograms depicting the monosaccharide composition of standard (A), crude (PLP) (B), and fractionated polysaccharides including PLF1 (C), PLF2 (D), and PLF3 (E).

Further fractionation of PLP on a DEAE Sepharose FF column produced three fractions: PLF1 yielded 26.3% (distilled water), PLF2 yielded 55.4% (0.5 M NaCl) and PLF3 yielded 18.2% (1.5 M NaCl) (Figure 1). All three fractions exhibited various levels of components, including neutral sugars (67.8%–84.1%), sulfates (0.9%–19.78%) and uronic acids (1.6%–4.2%) (*p* < 0.05). Upon sugar analysis, the PLP structure exhibited various monosaccharides, including glucose (55.5%), galactose (19.5%) and arabinose (13.8%) as the predominant units (Figure 2A) (*p* < 0.05). Other monosaccharides, including rhamnose and mannose, were also present in the PLP structure. The levels of the monosaccharides in PLF1 differed from those of the PLP polysaccharide. In particular, glucose increased sharply to 70.8%, while galactose and arabinose decreased to 13.3% and 6.3%, respectively (Figure 2B) (*p* < 0.05). In contrast, the analysis of monosaccharides in PLF2 and PLF3 revealed important changes in the sugar levels of the polysaccharide chains, with galactose (36.9%–45.0%) being the most common sugar found (Figure 2C,D).

### 3.2 Molecular Properties of Fucoidans

**FIGURE 3:**
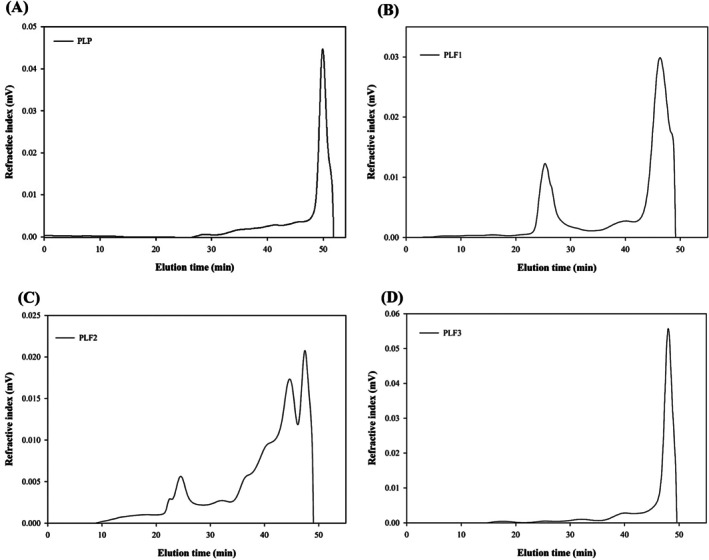
Profiles of refractive index (RI) on a TSK G5000PW column illustrating the characteristics of PLP, PLF1, PLF2, and PLF3 polysaccharides.

Figure 3 illustrates the refractive index (RI) profiles of all polysaccharides as they were eluted in the SEC column. In (Figure 3A)), a wide RI profile was obtained for PLP polysaccharide. While PLPF1 (Figure 3B) showed a fairly uniform size, the RI profiles of the PLF1 (Figure 3C) and PLF2 (Figure 3D) molecules showed a mix of different polysaccharide molecules with varying sizes. Molecular properties of the isolated polysaccharides were characterized using the MALLS system, revealing a high molecular weight (*M*_w_) of 881.8 × 10^3^ g/mol and a molecular size (*R*_g_) of 82.5 nm for PLP (Table 2). The polysaccharide molecules in PLF2 were found to be much bigger, with a molecular weight of 918.9 × 10^3^ g/mol. In contrast, the polysaccharide molecules in PLF1 and PLF3 were observed to be much smaller, with a molecular weight of 556.8 and 488.7 × 10^3^ g/mol, respectively. The *R*_g_ values for fractionated polysaccharides ranged from 59.1 to 72.1 nm. The *R*_g_ serves as a metric to express the size of a polysaccharide. In mathematical terms, it is expressed as the root mean square average of the distances of all molecular components from the center of the molecule. The PLP and PLF3 exhibited the highest *SV*_g_ values of 1.63 and 1.94 cm^3^/g, respectively. This value is inversely correlated with molecular compactness and suggests a greater molecular expansion compared to other polysaccharides (Bahramzadeh et al. 2019). In contrast, the polysaccharide molecules of PLF1 and PLF2 were estimated to have higher density and structural compactness, with *SV*_g_ values of 0.93 and 0.64 cm^3^/g, respectively.

### 3.3 Immune Cell Activation Capacity

#### 3.3.1 Stimulation of RAW264.7 Cells by PLF2

**FIGURE 4:**
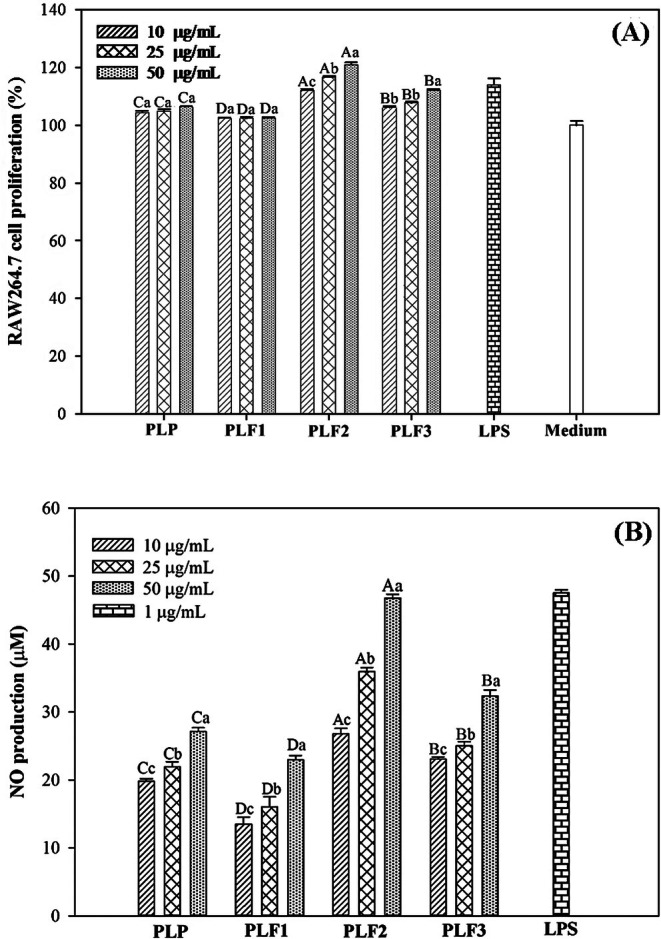
Effects of crude and fractionated polysaccharides (PLP, PLF1, PLF2 and PLF3) on cell proliferation (A) and nitric oxide production (B) in RAW264.7 macrophage cells. The cells were cultured in RPMI‐1640 medium with 10% FBS in a 96‐well microplate (100 μL volume, 1 × 10^4^ cells/well) and treated with various polysaccharides or LPS (positive control; 1 μg/mL). Letters a, b, c, d denote significant differences (*p* < 0.05) between polysaccharide concentrations, while uppercase letters A, B, C, D indicate significant differences (*p* < 0.05) between different polysaccharides at each concentration.

When crude and purified polysaccharides were incubated with RAW264.7 cells, no cytotoxicity was observed in the concentration range of 10 to 50 μg/mL, as shown in Figure 4A. Remarkably, these polysaccharides not only were nontoxic but also exhibited the ability to increase the proliferation of macrophage cells. Among them, PLF2 showed the highest activity, surpassing 20% (*p* < 0.05).

In response to stimulation, macrophages can release a significant level of chemokines and other proinflammatory mediators to regulate their function and enhance the acquired immune system (Schultze and Schmidt 2015). A main sign that macrophages are activated is the production of nitric oxide (NO), which helps protect against harmful germs and the growth of cancer cells (Borazjani et al. 2017). After adding different amounts of both crude and purified polysaccharides (10 to 50 μg/mL) to RAW264.7 cells, the macrophages started to release different amounts of NO, as shown in Figure 4B. A dose‐dependent elevation in NO quantity was obtained in cell medium after PLP treatment (28 μmol at 50 μg/mL) (*p* < 0.05). In contrast, RAW264.7 cells incubated with PLF1 exhibited a minimal response (24 μmol of NO). The highest NO release was observed in RAW264.7 cells incubated with PLF2, reaching 47 μmol (*p* < 0.05). This was comparable to the NO amount induced by bacterial LPS (48 μmol). Although NO release from RAW264.7 cells stimulated by PLF3 was superior to those of PLP and PLF1, it was inferior to that of PLF2. In accordance with prior discussions, it is important to emphasize that the efficacy of a polysaccharide in stimulating cells is contingent on its distinct structural characteristics. This ability can differ significantly from one polysaccharide to another. For example, the inflammatory response induced in macrophages by fucoidan from *Sargassum angustifolium* was found to be closely associated with sulfates esterified to the polysaccharide chain (Borazjani et al. 2018). Conversely, the immunostimulatory effects of a galactan sourced from *Opuntia polyacantha* were more pronounced in lower molecular weights (Schepetkin et al. 2008). Structure–activity relationships (SAR) of polysaccharides are not always conclusive, as it was not for the sulfated polysaccharide of *M. spicatum* in which no recognizable correlations were observed between the macrophage‐stimulating capacities of the most active polysaccharide and the presence of sulfate groups or the molecular characteristics (Alavi et al. 2020). Basically, the major findings of the reported studies highlight the importance of the presence of sulfate groups and optimal size for polysaccharides to exhibit a certain degree of biological activity, as all PLF1, PLF2, and PLF3 polysaccharides did here (Ferreira et al. 2015). However, no distinct structural features could be assigned to the exceptional cell activation capacities of the polysaccharides examined in the present study, implying that the enhanced stimulation potential of PLF2 is likely influenced by a combined effect of several structural characteristics rather than being attributed to a single structural characteristic. The current findings showed that even though PLP and PLF3 polysaccharides had more sulfates added to them than PLF2, this did not lead to better cell activation. In terms of molecular weight, the polysaccharide with the highest molecular weight, PLF2, had the strongest stimulation effect; however, this was not true for PLP, indicating that the bioactivity of PLF3 polysaccharides depends on more than just one structural characteristic (Bahramzadeh et al. 2019). Nonetheless, it is necessary to state that the position of sulfate esters on polysaccharide chains could explain the variations of cell activation capacities among different polysaccharides, the role of which should be further investigated in a systematic experimental study (Tabarsa, Han, et al. 2012; Tabarsa, Lee, and You 2012).

**FIGURE 5:**
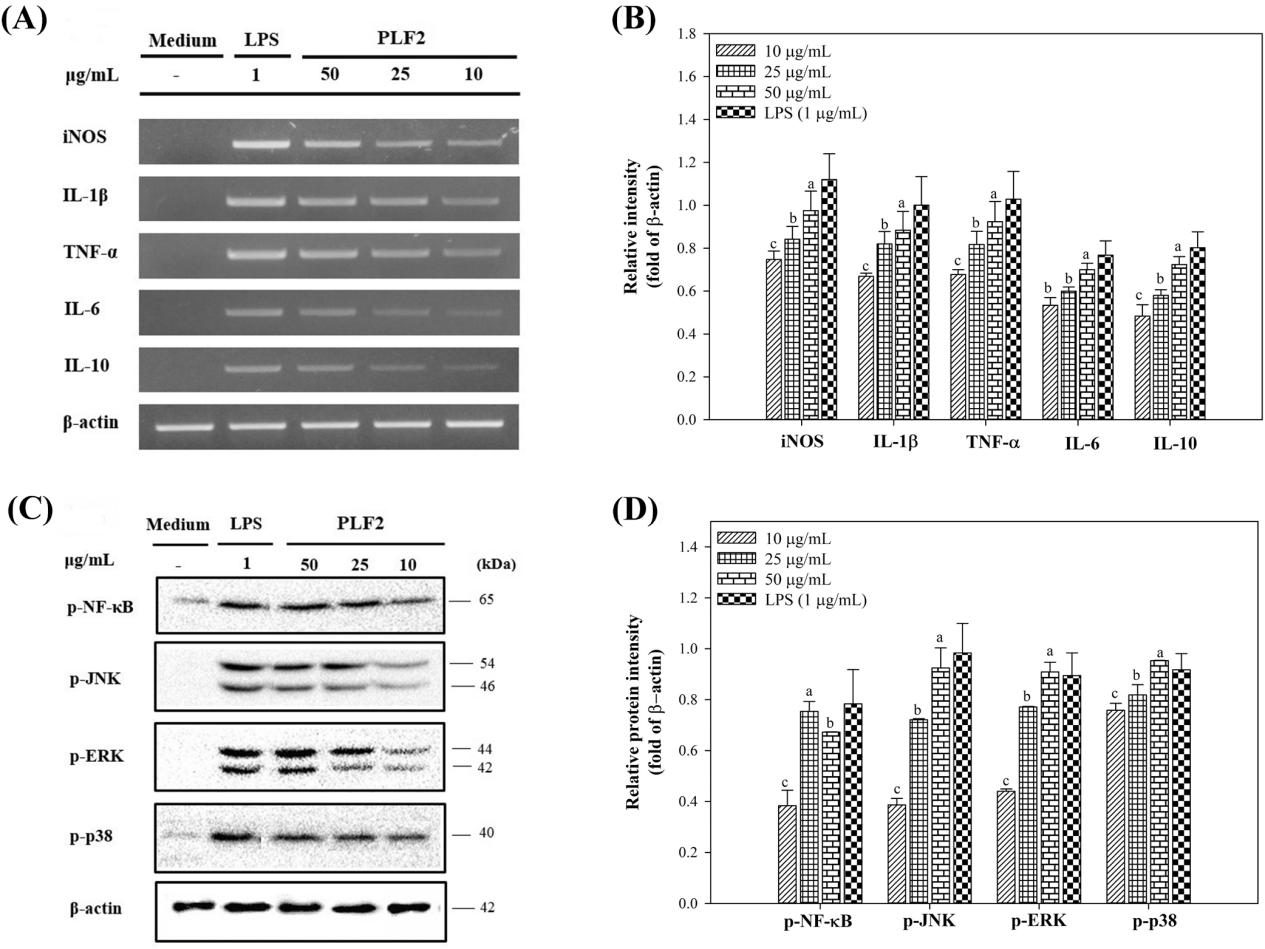
The mRNA expression of iNOS, IL‐1β, TNF‐α, IL‐6, and IL‐10 (A and B) as well as the expression of p‐NF‐κB, p‐JNK, p‐ERK, and p‐p38 proteins (C and D) in RAW264.7 macrophage cells activated by PLF2. Letters a, b, c, d denote significant differences (*p* < 0.05) between polysaccharide concentrations.

Given the ability of isolated polysaccharides, particularly PLF2, to enhance the growth and stimulation of RAW264.7 cells in releasing NO above the normal levels, the current study sought a more in‐depth investigation of the expression of major inflammatory mediators. Firstly, we examined the mRNA expression of inducible nitric oxide synthase (iNOS), which is accountable for catalyzing NO from L‐arginine. The results revealed a significant upregulation of iNOS mRNA after cell stimulation with PLF2 (Figure 5A,B) (*p* < 0.05). PLF2 induced transcription of main cytokine mRNAs of IL‐1β, TNF‐α and IL‐6 as opposed to sample‐free cells serving as the negative control (Figure 5A,B) (*p* < 0.05). These pro‐inflammatory cytokines are fundamental for the immune system and regulation of inflammation in the human body; they induce inflammation and play roles in fever induction, apoptosis, acute phase response, and immune regulation, but their dysregulation leads to chronic inflammatory diseases (Chen et al. 2017). Additionally, production of anti‐inflammatory mediators was investigated, revealing significant synthesis of IL‐10 mRNA (*p* < 0.05). This suggested a concurrent suppressive effect of PLF2 on disproportionate production of pro‐inflammatory cytokines and subsequent adverse symptoms. So far, the results have shown that PLF2 can act as an outside trigger for RAW264.7 cells, causing a significant pro‐inflammatory reaction. The subsequent step involved investigating the mechanism behind this response through potential signaling pathways.

**FIGURE 6:**
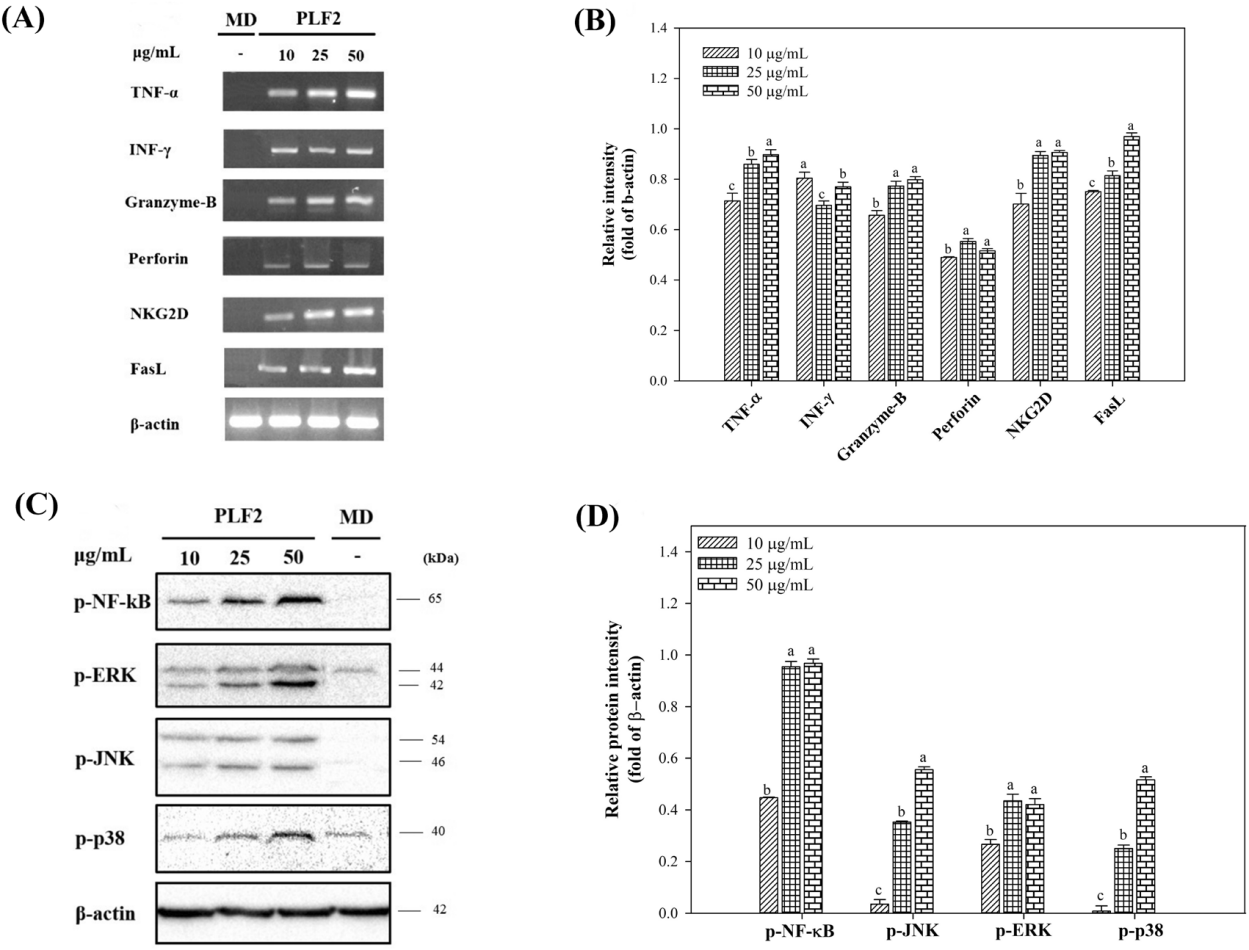
The mRNA expressions of TNF‐α, INF‐γ, granzyme‐B, perforin, NKG2D, and FasL in NK‐92 cells stimulated with PLF2 (A, B). Phosphorylation of NF‐κB, JNK, ERK, and p38 proteins in NK‐92 cells stimulated with the fraction with PLF2 (C, D). MD corresponds to cells incubated in a polysaccharide‐free medium. Letters a, b, c, d denote significant differences (*p* < 0.05) between polysaccharide concentrations. β‐Actin was used as control.

Nuclear factor kappa‐B (NF‐κB) and mitogen‐activated protein kinase (MAPKs) have been established as two main signaling pathways to convey the extracellular signals to the cell nuclei, thereby initiating the expression of the iNOS gene. Thus, Western blot analysis was employed to detect the phosphorylated NF‐κB, JNK1/2, ERK1/2, and p38 proteins in PLF2‐treated RAW264.7 cells (Figure 5C,D). As presented in Figure 6A,B,, identical to LPS‐stimulated cells, PLF2 efficiently initiated the production of phosphorylated NF‐κB in RAW264.7 cells, suggesting that the NF‐κB pathway served as the predominant signaling pathway driving the stimulation of macrophages induced by PLF2. NF‐κB is a ubiquitous transcription factor with a pivotal role in driving macrophage activation by transcribing inflammatory mediators (*p* < 0.05) (Liu et al. 2017). Additionally, the levels of phosphorylated ERK1/2 and JNK1/2 in RAW264.7 cells activated by PLF2 showed a clear and increasing trend based on the amount used (*p* < 0.05) (Figure 6A,B). The concentration of phosphorylated p38 (p‐p38), a protein kinase engaged in the control of TNF‐α gene expression, exhibited a significant elevation following the addition of PLF2 (Shen et al. 2017). In summary, these results suggested that PLF2 might have triggered the production of inflammatory substances in RAW264.7 macrophage cells by activating signaling pathways that include NF‐κB and MAPKs.

#### 3.3.2 Activation of NK‐92 Natural Killer Cells by PLF2

NK cells arise from a shared progenitor with T and B cells, forming a subset of lymphocytes. However, characterized by their function in the innate immune system, NK cells exhibit a prompt and versatile response to a diverse array of pathological stimuli. Therefore, we assessed the potential stimulation of NK‐92 cells by PLF2 by examining the synthesis of TNF‐α, IFN‐γ, granzyme‐B, perforin, NKG2D, and FasL. Figure 6A,B revealed that 10–50 μg/mL of PLF2 substantially upregulated the mRNA expressions of TNF‐α and IFN‐γ (*p* < 0.05). The release of TNF‐α and IFN‐γ cytokines by NK cells is crucial in regulating a range of potent inflammatory responses. These responses encompass the stimulation of macrophages and dendritic cells, the initiation of antiviral effects, and the swift mobilization of immune cells toward target organs (Lin et al. 2019). Beyond modulation of cytokine expressions, NK cells, once activated, execute a direct defense mechanism that involves the proactive approach of NK cells toward their target cells, where perforins contact the cell membrane, facilitating the creation of an aqueous channel. Through this channel, the granzyme gains access, initiating the apoptosis process (Lin et al. 2019). Likewise, in the present work, the introduction of PLF2 into the culture medium resulted in an up‐regulation of mRNA expressions for granzyme‐B and perforin, suggesting the capability of PLF2 to initiate direct toxicity in NK‐92 cells (*p* < 0.05). Similarly, the enhanced mRNA expressions of apoptosis‐inducing ligand (FasL) and the activating receptor NKG2D in the stimulated NK‐92 cells point to the activation of mechanisms independent of perforin. These mechanisms involve death receptor‐induced apoptosis and the engagement of activating receptors (Chua et al. 2004). Subsequent experiments aimed to shed light on the pivotal signaling pathways involved in the stimulation of NK‐92 cells by PLF2. Following a 24‐h incubation period with PLF2, a consistent and progressive increase in the amounts of phosphorylated NF‐κB (p‐NF‐κB) was observed when compared to cells left untreated (Figure 6C,D). Simultaneously, the levels of phosphorylated MAPK proteins, encompassing p‐ERK, p‐JNK, and p‐p38, exhibited elevation in PLF2‐activated NK‐92 cells (Figure 6C,D). These comprehensive findings strongly indicated that PLF2 possesses the capability to activate NK‐92 cells via the concerted engagement of both NF‐κB and MAPKs signaling pathways.

### 3.4 Structural Elucidation of PLF2

Methylation reaction was employed on PLF2 to elucidate the glycosidic linkages constructing its structure. The profile of partially methylated alditol acetates (PMAAs) disclosed that the predominant constituents of PLF2 structure were galactose‐based derivatives, including 1,3,5‐tri‐*O*‐acetyl‐D‐galactitol, 1,4,5‐tri‐*O*‐acetyl‐D‐galactitol, 1,5,6‐tri‐*O*‐acetyl‐D‐galactitol, 1,3,4,5‐tetra‐*O*‐acetyl‐D‐galactitol, 1,3,5,6‐tetra‐*O*‐acetyl‐D‐galactitol, and 1,3,4,5,6‐penta‐*O*‐acetyl‐D‐galactitol (Appendix S1, Table 3). This finding suggests the presence of specific glycosidic linkages, namely 1,3‐Gal*p*, 1,4‐Gal*p*, 1,6‐Gal*p*, 1,3,4‐Gal*p*, 1,3,6‐Gal*p*, and 1,3,4,6‐Gal*p*, within the PLF2 structure. Residues derived from arabinose were identified as the second most prevalent in the structure. The detected arabinose derivatives included 1,4‐di‐*O*‐acetyl‐D‐arabinitol, 1,2,5‐tri‐*O*‐acetyl‐D‐arabinitol, 1,3,5‐tri‐*O*‐acetyl‐D‐arabinitol, 1,4,5‐tri‐*O*‐acetyl‐D‐arabinitol, and 1,2,4,5‐tetra‐*O*‐acetyl‐D‐arabinitol. These derivatives correspond to glycosidic linkages of 1‐Ara*f*, 1,2‐Ara*p*, 1,3‐Ara*p*, 1,4‐Ara*p*, and 1,2,4‐Ara*p*. The identification of 1,5‐di‐*O*‐acetyl‐D‐glucitol, 1,2,5‐tri‐*O*‐acetyl‐D‐glucitol, and 1,4,5‐tri‐*O*‐acetyl‐D‐glucitol products revealed the occurrence of specific glycosidic linkages in the PLF2 structure. These linkages include 1‐Glc*p*, 1,2‐Glc*p*, and 1,4‐Glc*p*. The presence of 1,5‐di‐*O*‐acetyl‐D‐rhamnitol, 1,2,5‐tri‐*O*‐acetyl‐D‐rhamnitol, 1,2,4,5‐tetra‐*O*‐acetyl‐D‐rhamnitol, 1,5‐di‐*O*‐acetyl‐D‐mannitol, 1,4,5‐tri‐*O*‐acetyl‐D‐mannitol, and 1,5‐di‐*O*‐acetyl‐D‐xylitol derivatives showed the occurrence of 1‐Rha*p*, 1,2‐Rha*p*, 1,2,4‐Rha*p*, 1‐Man*p*, 1,4‐Man*p*, and 1‐Xyl*p* linkages in PLF2 structure (Table 3).

**TABLE 3:** The analysis of glycosidic linkages of PLF2 purified from *P. lucens* .

Glycosidic linkage	Methylation	PLF2
Xyl*p*‐(1→	1,5‐di‐*O*‐acetyl‐1‐deuterio‐2,3,4‐tri‐*O*‐methyl‐D‐xylitol	2.81
Ara*f*‐(1→	1,4‐di‐*O*‐acetyl‐1‐deuterio‐2,3,5‐tri‐*O*‐methyl‐D‐arabinitol	4.93
→2)‐Ara*p*‐(1→	1,2,5‐tri‐*O*‐acetyl‐1‐deuterio‐3,4‐di‐*O*‐methyl‐D‐arabinitol	1.72
→3)‐Ara*p*‐(1→	1,3,5‐tri‐*O*‐acetyl‐1‐deuterio‐2,4‐di‐*O*‐methyl‐D‐arabinitol	5.79
→4)‐Ara*p*‐(1→	1,4,5‐tri‐*O*‐acetyl‐1‐deuterio‐2,3‐di‐*O*‐methyl‐D‐arabinitol	1.91
→2,4)‐Ara*p*‐(1→	1,2,4,5‐tetra‐*O*‐acetyl‐1‐deuterio‐3‐mono‐*O*‐methyl‐D‐arabinitol	3.43
Rha*p*‐(1→	1,5‐di‐*O*‐acetyl‐1‐deuterio‐2,3,4‐tri‐*O*‐methyl‐D‐rhamnitol	2.90
→2)‐Rha*p*‐(1→	1,2,5‐tri‐*O*‐acetyl‐1‐deuterio‐3,4‐di‐*O*‐methyl‐D‐rhamnitol	1.90
→2,4)‐Rha*p*‐(1→	1,2,4,5‐tetra‐*O*‐acetyl‐1‐deuterio‐3‐mono‐*O*‐methyl‐D‐rhamnitol	4.22
Man*p*‐(1→	1,5‐di‐*O*‐acetyl‐1‐deuterio‐2,3,4,6‐tetra‐*O*‐methyl‐D‐mannitol	4.67
→4)‐Man*p*‐(1→	1,4,5‐tri‐*O*‐acetyl‐1‐deuterio‐2,3,6‐tri‐*O*‐methyl‐D‐ mannitol	2.12
Glc*p*‐(1→	1,5‐di‐*O*‐acetyl‐1‐deuterio‐2,3,4,6‐tetra‐*O*‐methyl‐D‐glucitol	10.67
→2)‐Glc*p*‐(1→	1,2,5‐tri‐*O*‐acetyl‐1‐deuterio‐3,4,6‐tri‐*O*‐methyl‐D‐glucitol	3.41
→4)‐Glc*p*‐(1→	1,4,5‐tri‐*O*‐acetyl‐1‐deuterio‐2,3,6‐tri‐*O*‐methyl‐D‐glucitol	3.67
→3)‐Gal*p*‐(1→	1,3,5‐tri‐*O*‐acetyl‐1‐deuterio‐2,4,6‐tri‐*O*‐methyl‐D‐galactitol	6.68
→4)‐Gal*p*‐(1→	1,4,5‐tri‐*O*‐acetyl‐1‐deuterio‐2,3,6‐tri‐*O*‐methyl‐D‐galactitol	13.97
→6)‐Gal*p*‐(1→	1,5,6‐tri‐*O*‐acetyl‐1‐deuterio‐2,3,4‐tri‐*O*‐methyl‐D‐galactitol	11.57
→3,4)‐Gal*p*‐(1→	1,3,4,5‐tetra‐*O*‐acetyl‐1‐deuterio‐2,6‐di‐*O*‐methyl‐D‐galactitol	2.40
→3,6)‐Gal*p*‐(1→	1,3,5,6‐tetra‐*O*‐acetyl‐1‐deuterio‐2,4‐di‐*O*‐methyl‐D‐galactitol	7.52
→3,4,6)‐Gal*p*‐(1→	1,3,4,5,6‐penta‐*O*‐acetyl‐1‐deuterio‐2‐mono‐*O*‐methyl‐D‐galactitol	2.94

**FIGURE 7:**
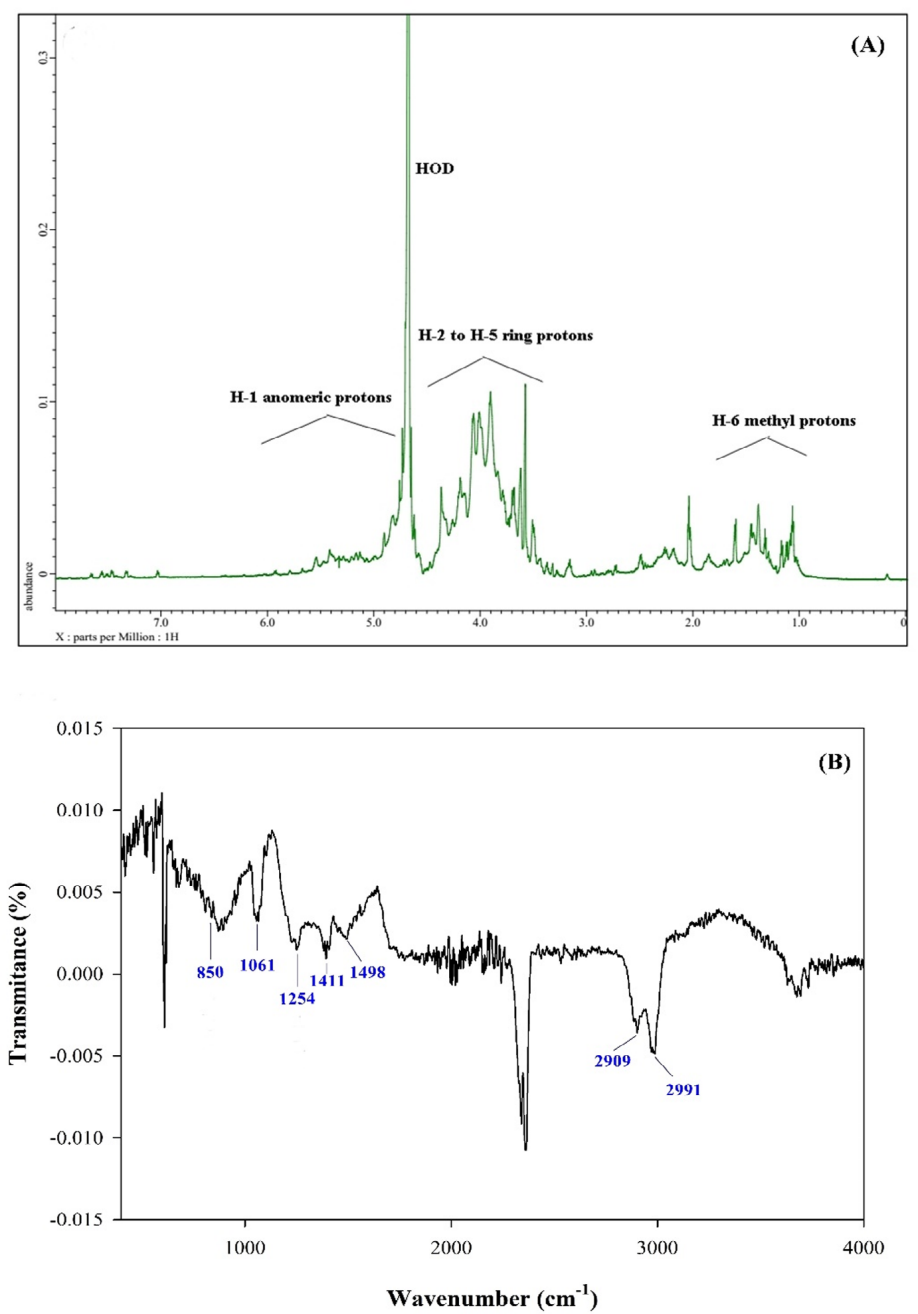
^1^H NMR spectrum of PLF2 dissolved in D_2_O and scanned at 50°C (A) and ATR‐FTIR spectrum of PLF2 (B).

In complement to the conducted glycosidic linkages analysis, the ^1^H NMR spectrum of PLF2 polysaccharide was obtained, revealing a spectrum with poor resolution and overlapping signals, emphasizing the complicated nature of the polysaccharide linkages (Figure 7A). Despite the challenges in obtaining a well‐resolved NMR spectrum, different proton spin systems were observed as follows: anomeric protons (4.7–6.0 ppm), H2‐5 ring protons (4.6–3.2 ppm) and H6 methyl protons (1.1–1.8 ppm) were observable in the ^1^H NMR spectrum. Figure 7B depicts the FT‐IR spectrum (ATR mode) of PLF2 polysaccharide. Distinctive absorption bands, characteristic of polysaccharides, were observed in the spectrum of PLF2. Within the wide scanned range of 400–4000 cm^−1^, particularly between 800–1200 cm^−1^ and 3400 cm^−1^, strong characteristic signals were observed for PLF2 (Coimbra et al. 2002). Consistent with sulfated polysaccharides, distinct peaks at 850 cm^−1^ (stretching vibration of S = O in sulfate) and 1250 cm^−1^ (bending vibration of C‐O‐S in sulfate with an axial position) further confirmed the substitution of sulfate esters on PLF2 polysaccharide chains (Tabarsa et al. 2020).

## 4 Conclusions

PLF2, a galactose‐rich polysaccharide isolated from *P. lucens* , exhibited the ability to activate RAW265.7 and NK‐92 cells by engaging the NF‐κB and MAPKs signal pathways. Consequently, this activation led to the synthesis of NO and various cytokines including TNF‐α, IL‐1β, IL‐6, and IL‐10 within murine macrophage cells. Additionally, in natural killer cells, PLF2 induced the synthesis of mediators such as TNF‐α, IFN‐γ, GrB, perforin, NKG2D, and FasL. The potential of the examined polysaccharide to activate cells may be associated with factors such as the size of the molecule and its conformation. The credibility of this correlation should be rigorously examined through systematic investigations in future studies. Besides, the high heterogeneity of the glycosidic linkages presented in the PLF2 polysaccharide immensely restricted the elucidation of residual sequences of the polysaccharide using NMR spectroscopy. Thus, well‐resolved spectra could be obtained by implementing chemical and enzymatic approaches to produce low molecular weight fragments prior to NMR analysis.

## Author Contributions

**Rongan Cao:** conceptualization (equal). **Mehdi Tabarsa:** conceptualization (equal), writing – original draft (equal), writing – review and editing (equal). **SangGuan You:** formal analysis (equal). **Fatemeh Noormand Chaloshtori:** formal analysis (equal). **Jiamiao Zhang:** formal analysis (equal). **Kou Fang:** formal analysis (equal). **Seyedeh Zahra Bathaie:** data curation (equal).

## Conflicts of Interest

The authors declare no conflicts of interest.

## Supporting information


**Appendix S1:** fsn370692‐sup‐0001‐supinfo.docx.

## Data Availability

The data of the present study are available in response to request.
