# Supplementary material for: Sulfated Polysaccharides of Potamogeton lucens as a Promising Immunostimulatory Agent in Activation of RAW264.7 and NK Cells
Source: Food Sci Nutr. 2025 Aug 8;13(8):e70692. doi: 10.1002/fsn3.70692 (PMC12334546; doi:10.1002/fsn3.70692)
Supplement: Supplementary file 1 — Appendix S1: fsn370692‐sup‐0001‐supinfo.docx. [file FSN3-13-e70692-s001.docx]

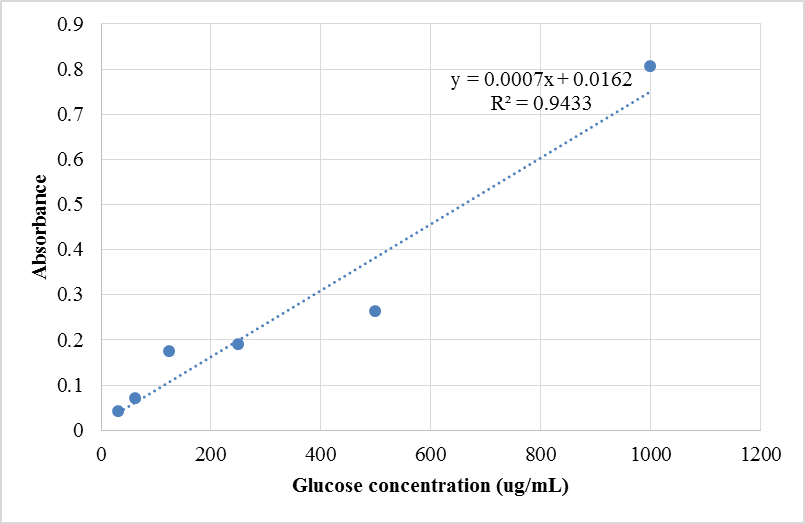


**Sup. 1.** Standard curve of glucose used for determination of neutral sugar content.

**
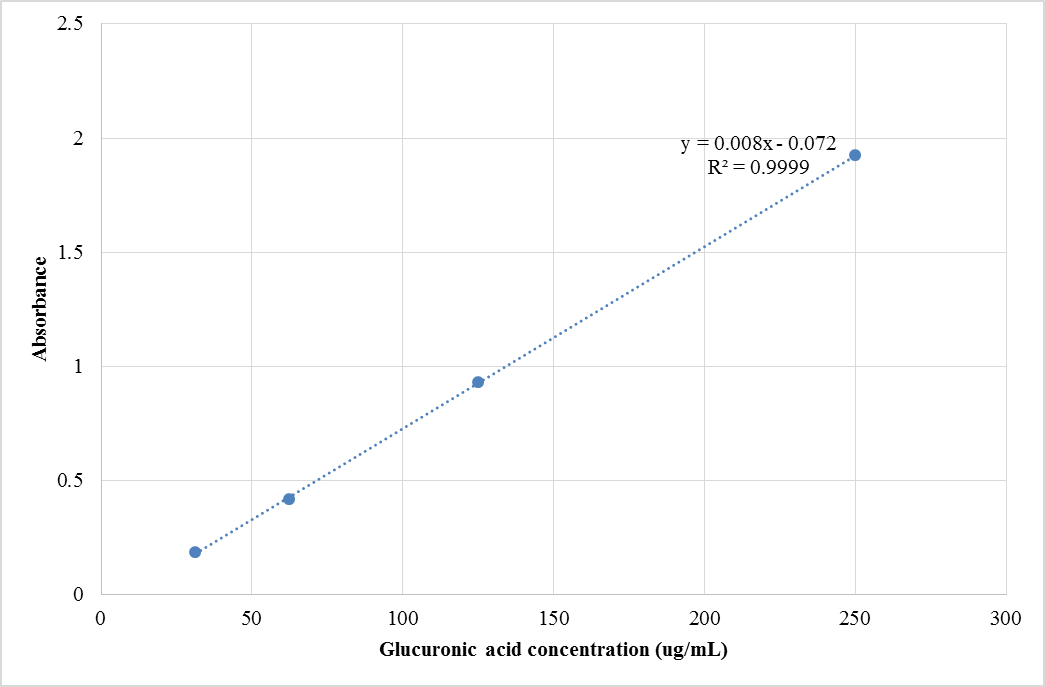
**

**Sup. 2.** Standard curve of glucuronic acid used for determination of uronic acid content.

**
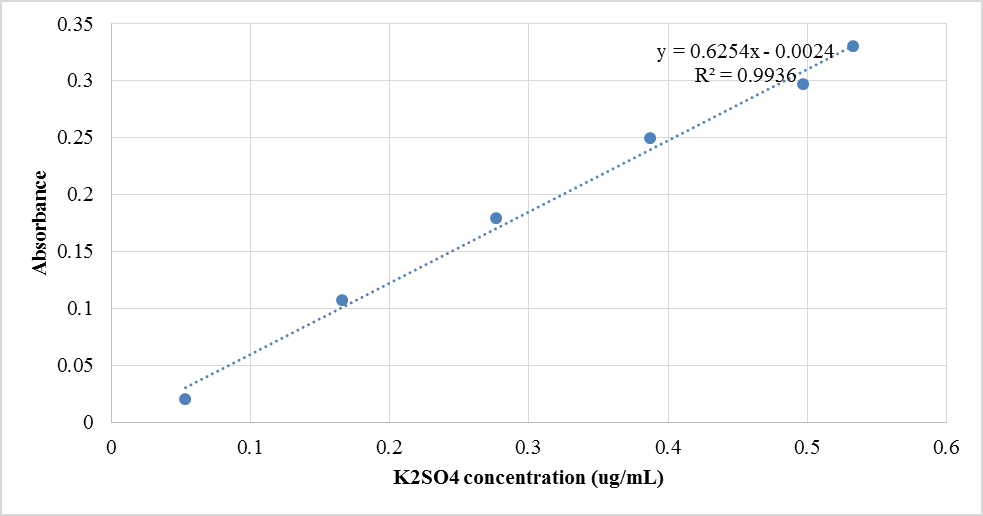
**

**Sup. 3.** Standard curve of K_2_SO_4_ used for determination of sulfate content.

**
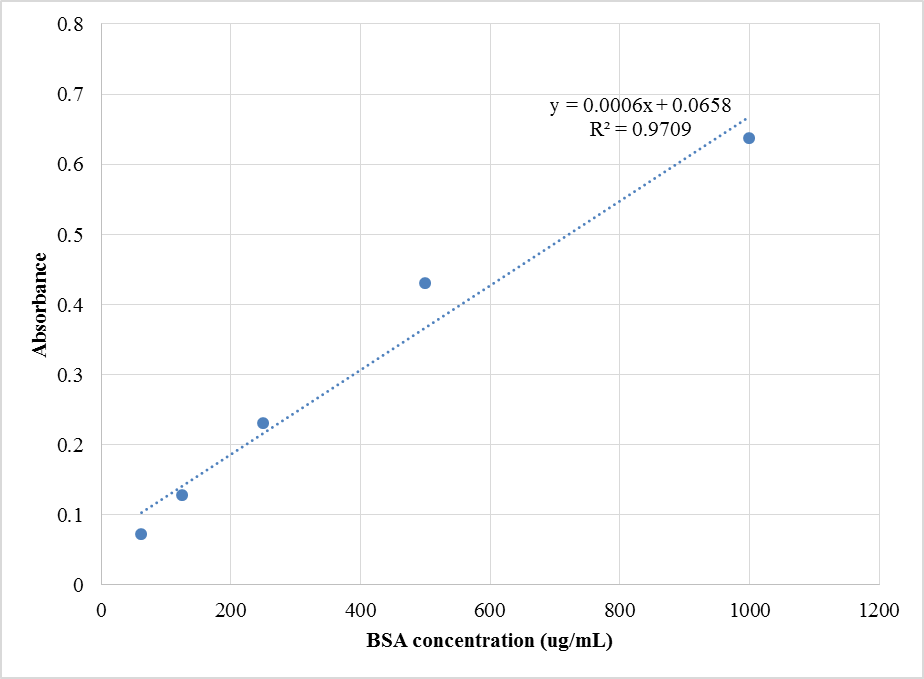
**

**Sup. 4.** Standard curve of BSA used for determination of protein content.


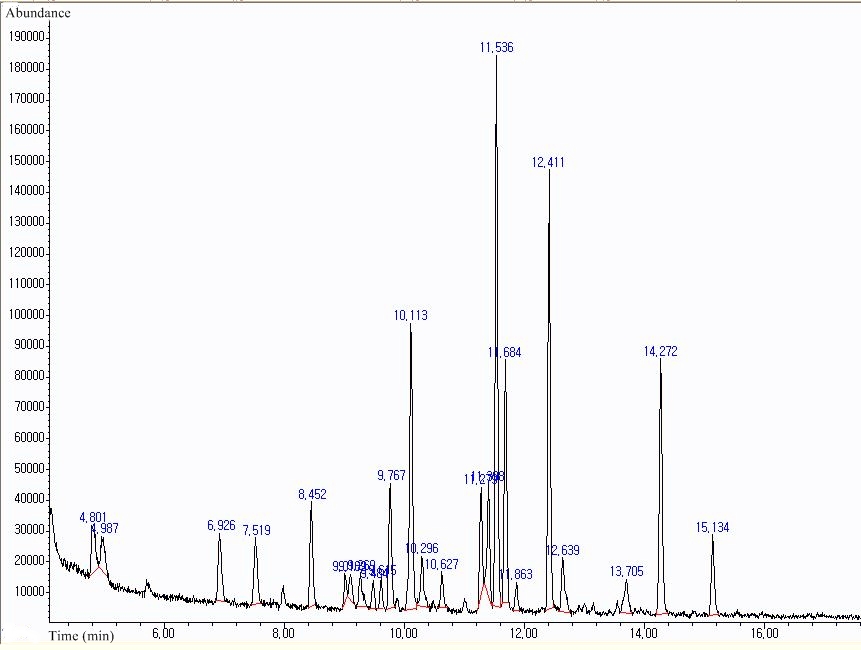


**Sup. 5.** The GC chromatogram of PMAAs prepared from PLF2 polysaccharide.
